# Social Networks Addiction (SNA-6) – Short: Validity of Measurement in Mexican Youths

**DOI:** 10.3389/fpsyg.2021.774847

**Published:** 2022-01-12

**Authors:** Edwin Salas-Blas, César Merino-Soto, Berenice Pérez-Amezcua, Filiberto Toledano-Toledano

**Affiliations:** ^1^Instituto de Investigación de Psicología, Universidad de San Martín de Porres, Lima, Peru; ^2^Centro de Investigación Transdisciplinar en Psicología, Universidad Autónoma del Estado de Morelos, Cuernavaca, Mexico; ^3^Unida de Investigación en Medicina Basada en Evidencias, Hospital Infantil de México Federico Gómez, Mexico City, Mexico; ^4^Unidad de Investigación Sociomédica, Instituto Nacional de Rehabilitación Luis Guillermo Ibarra Ibarra, Mexico City, Mexico

**Keywords:** random intercept model, social networks, addiction, validity, adolescents, Mexican

## Abstract

The excessive use of social networks needs to be addressed, and this phenomenon needs to be measured for the purpose of evaluation, prevention, and intervention among adolescents and young people. The objective of the study was to adapt and psychometrically validate the Brief Scale of Addiction to Social Networks (SNA-6) among Mexican adolescents and young adults. The participating sample consisted of 2,789 students from 6 public educational campuses in Cuernavaca (Morelos, Mexico). Data collection was carried out through a web platform to strictly maintain anonymity, voluntary participation, and confidentiality. Data analysis first focused on the detection of possible response biases (random intercept model and careless/insufficient effort), the quality of the response structure partial credit model (PCM), dimensionality (CFA and invariance), and the relationship with external variables. It was found that when the range of efficient response options was limited to less than five, reliability was high (0.91), and unidimensionality was maintained. Response biases slightly affected the dimensional structure of the instrument. Measurement invariance reached scalar invariance in the sex, age, and campus groups. The association with sensation seeking and depression, controlling for sex and age covariates, was statistically significant, small, and theoretically consistent. Implications of the results are discussed.

## Introduction

A Digital 2021 report carried out by We Are Social and Hootsuite indicates that the number of users of social networks (SNs) in the world continues to increase vigorously,^1^ maintaining that by January 2021, there were 4,200 million users, which is approximately 53% of the world population. This represents 13% growth over the previous year. In the Americas, the countries that have experienced the greatest growth are Mexico, the United States, and Brazil. The largest group of internet users are between 25 and 34 years old, followed by those between 18 and 24 years old. In both age groups, men spend more time networking. The average time invested in SNs globally is 2 h 25 min, but in Mexico, it is 3 h.

The development of information technologies, particularly smartphones, as well as the widespread use of the internet and its current low costs, are elements that could explain the increase in the use of virtual SNs, which are becoming increasingly attractive to consumers. However, SNs have positive aspects, such as allowing people to establish rapid communication over distances and being used in work or study situations. However, if SNs are not used properly, they could result in health problems (Salas, 2014; Loss et al., 2020) that are associated with certain risks and problematic behaviors (Turel and Serenko, 2012; Matute, 2016; Medrano et al., 2017; Valencia-Ortiz et al., 2021); at the extreme, SN use could result in dependency, addiction, or pathological behaviors (Andreassen et al., 2012; Guertler et al., 2014; Chóliz, 2016a; Echeburúa, 2016; Matute, 2016) that require intervention or the development of preventive programs (Chóliz and Marco, 2012; Marco and Chóliz, 2013; Chóliz, 2016b; Marcos, 2020).

The popularity and massive use of SNs peaked in the second decade of this century, and there is more interest in the problems that could arise from internet and SN excessive use, abuse, problematic use, pathological use, addiction or dependence, and this interest and concern has led scholars around the world to develop numerous studies in the theoretical and applied fields (Echeburúa and Gargallo, 2010; Tao et al., 2010; Turel and Serenko, 2012; Puerta-Cortés and Carbonell, 2013; Salas, 2014; Gil et al., 2015; Matute, 2016; Hussain and Griffiths, 2018; Cabero-Almenara et al., 2020a; Ruiz-Ruano et al., 2020; Lupano and Castro, 2021).

Many researchers have focused on measuring phenomena that do not have clear definitions or understanding of their similarities or differences (excessive use, problematic use, abusive use, addiction, risk of addiction, pathological or compulsive use of the internet and SNs), and related phenomena such as nomophobia (fear of missing out). These scales have been adapted and validated in various languages and parts of the world (Beranuy et al., 2009; Meerkerk et al., 2009; Lam-Figueroa et al., 2011; Andreassen et al., 2012; Karim and Nigar, 2014; Boubeta et al., 2015; Vilca and Vallejos, 2015; Fonsêca et al., 2018a,b; Peris et al., 2018; Sahin, 2018; Vallejos-Flores et al., 2018; da Veiga et al., 2019; Laconi et al., 2019; Cabero-Almenara et al., 2020a,b; García-Umaña and Córdoba, 2020; Santamaria and Vallejos-Flores, 2021). This broad conceptual, instrumental and contextual coverage suggests that interest in this topic has increased worldwide.

The SN Addiction questionnaire (SNA) (Escurra and Salas, 2014) was constructed with the diagnostic and statistical manual - 4 (DSM 4) criteria for the diagnosis of substance addiction. It has 24 items grouped into three dimensions: obsession with SNs, lack of personal control in the use of SNs, and excessive use of SNs (Escurra and Salas, 2014). Its validity has been replicated multiple times, with its factorial structure maintained in some studies (Medrano et al., 2017; Salazar-Concha et al., 2021) and varied in others. Bueno et al. (2019) found five dimensions, and others argue that the instrument is unidimensional (Fonsêca et al., 2018b; Salas-Blas et al., 2020). The latter structure motivated and justified the construction of the SNA-6 (Salas-Blas et al., 2020), but with a different theoretical basis (Griffiths, 2005a), called the *Components Model*, which has six dimensions. Within the SNA-6, each item represents one of these six components. The model of the components of addiction from a biopsychosocial perspective, according to Griffiths (1996, 2005a,b), is applicable to any addictive behavior, with and without substances, and is based on the following components: (a) *Salience*, which consists of the excessive behavior becoming the most important thing for the addicted person, because it dominates his or her thoughts, feelings and behaviors; (b) *Mood change*, which are subjective experiences resulting from the activity, such as ecstasy, numbing and escape from reality; (c) *Tolerance*, in which more time is dedicated to the activity; (d) *Abstinence*, which manifests itself in states of discomfort, trembling, irritability in the absence of the addictive activity, and which are only overcome when the activity is resumed; (e) *Conflicts*, generated between the addict and the people around him/her, such as friends, family, partner, work, school, etc.; (f) *Relapses*, tendency to repeatedly return to previous patterns after experiencing abstinence.

The six items of the brief version of the SNA (SNA-6), come from the original version of 24 items (Escurra and Salas, 2014), they were chosen by expert judges who first located to which of the components each of the 24 items theoretically belonged; once this first classification was done, they were asked to choose two items that they considered theoretically the most representative of each factor. These twelve items were then statistically analyzed to form the six most empirically representative. The items were formulated with the following characteristics: (a) in construct orientation, where the response in the high levels of the scale should correspond to high levels in the construct measured, (b) the use of the instrument is mainly oriented for group description and individual screening, (c) the content shows the temporal intensity of the behavior.

The purpose of this brief version is to facilitate complex research; the development of studies with different populations, both in size and in cultural variety; and studies in which several variables are related, both in predictive research models and in multicausal type. In this type of complex study, the use of short but valid instruments is necessary, as in the present investigation, which focuses on the covariation of SNA with external variables, such as sex, sensation seeking, and symptoms of depression.

Regarding the relationship between SN addiction and sex, there are contradictory results; some studies found that men obtain higher scores (Salas and Escurra, 2014; Müller et al., 2016; Bueno et al., 2019), others found that women have higher scores (Rodriguez and Fernandez, 2014; Peris et al., 2018), and still, others found no differences. A similar situation occurs when comparisons are made by age groups (Beranuy et al., 2009; Salas-Blas, 2019b; Loss et al., 2020); these contradictory data may vary by culture.

Although there is evidence of relationships between sensation seeking and some phenomena, such as risk behaviors, tobacco consumption, alcohol consumption, aggressiveness, antisocial behavior, and substance addictions (Lorca and Sanz, 2003; Nadal, 2008; Merino-Soto and Salas Blas, 2018), there is little research on its relationship with behavioral addictions and specifically, internet and SN addiction; some studies have found positive relationships of impulsivity, lack of control, and gratification seeking (which could also characterize the sensation-seeking) with addictions to the internet and SNs (Koo and Kwon, 2014; Zhang et al., 2015; Becoña, 2016; Clemente et al., 2019; Lupano and Castro, 2021). Based on this evidence and the conviction that other addictions have the same characteristics as substance addictions, that sensation seeking and addiction can be assumed to be positively correlated with SN addiction.

Regarding the relationship between depressive symptoms and addiction to SNs, there are many studies based on applying the first measure of addiction to the internet (Young, 1998), and the results consistently show positive correlations between the two phenomena (Zhang et al., 2015; Becoña, 2016; Błachnio et al., 2016; Hussain and Griffiths, 2018; Peris et al., 2018; Iovu et al., 2020). Therefore, it is expected that this study will find similar results. The study of dependence on technologies, including SNs, is increasing and relevant to many phenomena and activities in people’s lives. This work has methodological importance in validating a scale to measure addictions to SN in adolescents and young people in Mexican, across its wide territory and very large population. The effects of irrelevant responses to the construct, such as those *via* the careless/insufficient effort response (C/IE), are addressed. Research has shown such effects to be common (Faust et al., 2012; Meyer et al., 2013), and any such effects must be identified and addressed because they have been shown to affect the classification of subjects (Emons et al., 2007) and the assessment activities for the classification of subjects with possible behavioral dependencies. Additionally, there is an incremental effect of false positives (Faust et al., 2012; Meyer et al., 2013), as well as psychometric properties in general, such as dimensionality, internal structure, and reliability (Liu et al., 2010; Arias et al., 2020). These issues are particularly important and impactful for short scales because the accuracy of respondent classification is linked to the prevalence of careless/insufficient response effort (C/IE) and other factors (Emons et al., 2007). In this regard, there is a call to take C/IE into account in the field of addiction studies (Godinho et al., 2016; King et al., 2018), and the present study addresses this potential problem as the first step toward the main objective of the study.

Therefore, the objective of the present study is to adapt and psychometrically validate the Brief Questionnaire on Addiction to Social Networks (SNA-6) with adolescents and young Mexicans. For this, evidence of the internal structure (i.e., dimensionality, measurement invariance, and reliability; American Educational Research Association, American Psychological Association, and National Council on Measurement in Education, 2014) and associations with other variables were obtained.

Because there is an apparent weakness in the methodology for examining the psychometric performance of items in epidemiological and public health research (Olsen, 1998; Hagquist, 2019), and the SNA can potentially be in these research areas, the present study introduced the study of the quality of response options. Within this framework of methodological rigorousness, it was taken into account that external sources of information (i.e., constructs or external criteria) are drawing attention to give better and more supported conclusions about construct validity (e.g., see Taras and Kline, 2010; Ferrando et al., 2019; Ferrando and Lorenzo-Seva, 2019), so the item analysis included the analysis of association validity with external criterion. This has connections with construct validity at the level of specific items (Taras and Kline, 2010), with content validity (Koller et al., 2017), or with the emergence of differential relationships with external variables (Ferrando and Lorenzo-Seva, 2019).

The following hypotheses served as a framework to guide the interpretation of the results: first, the structure of the response options will show optimal characteristics (i.e., in agreement with Linacre guidelines; see Procedure section); second, the latent structure of the SNA-6 scores will be unidimensional; third, the internal structure properties will be invariant across gender, age, and campus groups; and fourth, the relationships of the SNA-6 with measures of depressive symptoms and sensation-seeking will theoretically converge (different from zero).

## Materials and Methods

### Participants

The population invited to participate comprised Mexican students at the upper secondary level between 15 and 19 years old who were selected to explore potential areas of intervention in subjects related to mental health. The selected population was from five campuses id Cuautla, Cuernavaca, Jiutepec, Temixco, and Tepoztlán in the state of Morelos (Mexico). The selected sample comprised adolescents who met the following inclusion criteria: enrolled as an upper secondary student in one of the five selected schools and providing parental authorization with the informed consent and assent of the participant. Data due to potential careless/insufficient effort responses were excluded.

The initial participating sample included 2,998 individuals (Table 1), distributed in the five schools as follows: 934, 371, 818, 347, and 528. For confidentiality, alphabetic campus names (A,B,C,D, and E campuses) were used to mask the true names. After excluding responses apparently due to careless/insufficient effort (see Results section), the effective sample for the study analysis was 2,789. The sex distribution was similar with respect to age (linear *χ^2^* = 3.11, *p* = 0.07, gamma = 0.04), campus (*χ^2^* = 73.75, *p* < 0.01, V Cramer = 0.13), marital status (*χ^2^* = 4.62, *p* < 0.05, Cramer V = 0.05), and semester (linear *χ^2^* = 2.79, *p* = 0.09, gamma = −0.05). However, there was a slight difference with respect to current employment status (*χ^2^* = 139.09, *p* < 0.01, Cramer V = 0.223).

**TABLE 1:** Distribution of demographic characteristics (*n* = 2,789)^a^.

	N	%
**Sex**		
Female	1406	50.4
Male	1383	49.6
**Campus**		
A	877	31.4
B	339	12.2
C	760	27.2
D	319	11.4
E	494	17.7
**Semester**		
2	1067	38.3
4	876	31.4
6	846	30.3
**Marital status**		
Single	2687	96.3
Married	22	0.8
Unmarried	80	2.9
**Work**		
Yes	945	33.9
No	1844	66.1
**Age**		
14	2	0.1
15	567	20.3
16	784	28.1
17	811	29.1
18	495	17.7
19	81	2.9
20	32	1.1
21	9	0.3
Missing	8	0.3

*^a^Sample with exclusion criterion used (C/IE responses).*

### Instruments

Because to the natural variability of sampling and in the relations between variables, and to avoid inducing internal structure validity based on previous reports without corroborating it in the actual participant sample (Merino-Soto and Calderón-De la Cruz, 2018; Merino-Soto and Angulo-Ramos, 2020), the internal structure of external construct measures (BSSS and CESD-7) was verified.

#### Brief Scale of Social Networks Addiction (SNA – 6) (Salas-Blas et al., 2020)

The SNA-6 was constructed from a previous version of 24 items and three dimensions published by Escurra and Salas (2014). From this initial study, the authors found three highly correlated dimensions, which were subsequently reduced in the current version, the SNA-6 (Salas-Blas et al., 2020). The SNA-6 measure evaluates addiction to SNs. The authors performed a content validation of the items using the Griffiths (2005b) component model, which proposes that addictions have six components (salience, mood change, tolerance, abstinence, conflicts, and relapse); in the SNA-6, there is an item that measures one of the factors. The unidimensionality of the SNA-6 was found in Peruvian adolescents (Salas-Blas et al., 2020) and corroborated in the validation in Brazil (Fonsêca et al., 2018b). The items are ordinally scaled in 5 response options (from 1 to 5), from *Not at all* to *Always*. Responses to the items are summed to obtain a total score. The Peruvian version (Salas-Blas et al., 2020) was used in the present study; and to ensure clarity of content, the Mexican co-author of this article verified the appropriateness of its content.

#### Brief Sensation Seeking Scale (Hoyle et al., 2002)

The BSSS evaluates the attribute of sensation seeking and is generally used to detect the risk of substance use in adolescents. It consists of eight items scaled in five points (from strongly disagree to strongly agree) and produces a unique score. The version for Latino adolescents was used (Stephenson et al., 2007) and was adapted for Peru (Merino-Soto and Salas Blas, 2018). In the present study, one of the Mexican authors of this manuscript checked the content for clarity and conceptual relevance for Mexican adolescents, and both aspects were found to be appropriate. Also, the unidimensionality of the instrument adjusted satisfactorily, weighted least squares means and variance (WLSMV)-*χ^2^* = 436.65, df = 20, *p* < 0.01; CFI = 0.993, RMSEA = 0.034, SRMR = 0.048, WRMR = 2.69, with factor loadings between 0.602 y 0.842; the internal consistency was ω = 0.87 (IC95% = 0.86, 88, se = 0.004).

#### Center for Epidemiological Studies Depression Scale – 7 (Herrero and Gracia, 2007)

The measurement of depression symptoms (dysphoric mood, poor motivation, concentration, pleasure and sleep) was taken from Santor and Coyne (1997) and adapted to the Mexican context. It consists of seven items oriented toward depression, except item 6 (item 6 needed recoding), scaled in four points (from never to always). For the present study, the one-dimensional model of the CESD-7 was not satisfactory (WLSMV-*χ^2^* = 994.57, df = 14, *p* < 0.01; CFI = 0.985, RMSEA = 0.077, SRMR = 0.101, WRMR = 4.866), mainly due to the low load of item 6 (factor load = 0.067, se = 0.02). Without this item, the fit improved substantially [WLSMV-*χ^2^* = 265.90, gl = 15, *p* < 0.01; CFI = 0.996, RMSEA = 0.027, SRMR = 0.055, WRMR = 2.839 (factor loads between 0.440 and 0.935)]. The internal consistency of the six items was ω = 0.87 (95% CI = 0.86, 88, se = 0.004).

#### Sociodemographic Information Sheet

A sheet was used to record information about age, sex, semester of study, marital or marital status, and work activity.

### Procedure

#### Data Collection

The questionnaire was administered electronically between April and May 2019. The administration of the survey was directed and supervised by the counselors of each campus, who received training in standardized procedures for administering the survey and resolving situations that may arise. The administration procedure and the order of presentation of the instruments to the participants were the same for each campus: informed consent, demographic questions, and instrument questions. The specific steps were as follows: the consent form was given to the parent or guardian to allow their children to participate in the study, and then, informed consent was provided by each student. Finally, the participants responded collectively in the computer center. The data collection was guided by the principles of the Helsinki Declaration and the Belmont Report in several ways: the anonymity of response, voluntary participation, freedom to withdraw, and confidentiality of information collected. To protect subject anonymity, no identifiable human data were retained. The participants were informed of their right to continue or end their participation at any moment. Finally, the importance of honest responses, careful attention to the instructions and contents of the items, and willingness to resolve doubts about filling out the survey were highlighted.

#### Ethical Considerations

This study is a part of the research project (HIM/2015/017/SSA.1207; “Effects of mindfulness training on psychological distress and quality of life of the family caregiver”) that was approved on December 16, 2014, by the Research, Ethics, and Biosafety Commissions of the Hospital Infantil de México Federico Gómez National Institute of Health in Mexico City. While conducting this study, the ethical rules and considerations for research with humans in Mexico (Sociedad Mexicana de Psicología, 2010) and those outlined by the American Psychological Association (2017) were carefully followed. All family caregivers were informed of the objectives and scope of the research and their rights in accordance with the Declaration of Helsinki (World Medical Association, 2013). The caregivers who agreed to participate in the study signed an informed consent letter. Participation in this study was voluntary and did not involve payment.

#### Analysis of Data

The analysis was performed in the following sequence: evaluation of response biases, item analysis, internal structure analysis, and relationship with other variables.

##### Potential Response Biases

The SNA was included in a survey of 11 dimensions measuring psychological, emotional, and attitudinal attributes, with 50 total items, but several of these items nested with other items, for a total of 143 effective items measuring demographic information and other constructs (e.g., sensation seeking). Therefore, this extension of the questionnaire can be associated with the generation of careless/insufficient effort responses (C/IE; Huang et al., 2015; Curran, 2016) especially in adolescents (Cornell et al., 2012; Jia et al., 2018). C/IE can be expressed in response patterns with extreme or little variability (Curran, 2016), and in this sense, and as a general measure of anomalous responses, the presence of multivariate outliers was first verified using distance *D*^2^ (Mahalanobis, 1936), an efficient method to detect possible random responses (Zijlstra et al., 2011). This control reduces the sensitivity of the covariance matrix and the emergence of model specification errors of latent variables due to outliers (Wilcox, 2012).

Because the data were treated categorically in most of the analyses, the detection of outliers was done within a non-parametric approach for non-continuous variables, which are considered discordant observations expressed in *extreme scores* (e.g., O + y G + scores; Zijlstra et al., 2007), which differ from the regular scoring pattern due to their infrequency. *G*_+_ is based on the paired comparison of items to obtain the number of errors according to Guttman (1944). The complementary use of both has been recommended (Zijlstra et al., 2007), but *G*_+_ alone or in combination with the Mahalanobis distance are effective measures for the detection of random responses (Zijlstra et al., 2011, 2013). For the procedures described in this analysis, the R *careless* (Yentes and Wilhelm, 2021) and *mokken* (van der Ark, 2012) programs were used.

##### Item Analysis

First, descriptive and distributional statistics for the items were obtained. For association analysis, non-parametric indices were chosen to maintain the mainly categorical treatment given to the SNA-6 items. Thus, correlational effect size indices were used (*Glass rank-biserial*, *eta-squared*, and *epsilon-squared*; Tomczak and Tomczak, 2014). Together, these indicators served to investigate the possible differentiation of the items in their relationships with other variables. Quantitatively, this adds support for validity. The R *langtest* (Mizumoto, 2015), *rcompanion* (Mangiafico, 2021), and *MVN* (Korkmaz et al., 2014) programs were applied. Second, to evaluate the structural properties of the SNA response option in detail, item response theory modeling was used.

##### Properties of Ordinal Scaling

The structural properties of the response options for each SNA item were evaluated using the suggested guidelines of Linacre (1999, 2002a) that the response categories (a) have more than 10 responses, (b) show a monotonic increase in the thresholds with the latent attribute score, (c) have a regular distribution, (d) have ordering thresholds, and (e) have step difficulties that advance at least 1.0 logits but less than 5.0 logits. The application of these properties required evaluating whether the items fit the partial credit model (PCM). Given the relative independence of the mean square statistics [Infit mean square (In-MSQ); outfit mean square (Out-MSQ)] from the sample size, these fit indicators were preferred over the accompanying statistical tests (t test for Outfit and Infit) (Smith et al., 2008). The R eRm program was used (Mair et al., 2021).

##### Internal Structure

We use the confirmatory factor analysis within the structural equations modeling (CFA-SEM) approach. The WLSMV estimator applied to the polychoric correlations between the items was used since it helps obtain psychometric parameters with greater precision for categorical and non-normal variables (Finney and DiStefano, 2013). In the framework of model comparison, a model with a method factor was included to estimate a possible variance associated with response patterns (Maydeu-Olivares and Steenkamp, 2018). For this, the random intercepts model was implemented (random intercepts factor analysis: RIFA; Maydeu-Olivares and Coffman, 2006). The usual specification for RIFA is to include a factor to all items (i.e., method factor), in addition to the substantive factors (in this study, a single SNA substantive factor), and set their factor loadings to 1 (to establish the equality between them). In the present study, the generalized random intercepts framework was implemented, in which it is specified that the effect of method variance varies in each item (Maydeu-Olivares and Steenkamp, 2018). This contrasts with the presumption of equality of the method variance in the items proposed in the original method (Maydeu-Olivares and Coffman, 2006). The models that presented close fit were those with the following degrees of fit: RMSEA ≤ 0.05, SRMR ≤ 0.05, and CFI ≥ 0.95; Maydeu-Olivares (2017). The reasonable adjustment was recognized when RMSEA ≤ 0.08, SRMR ≤ 0.08, and CFI ≥ 0.90 (Hu and Bentler, 1999). Possible adjustments to improve the model were examined using the statistical power and size of the parameter (i.e., correlated residual) and were implemented by the approach of Saris et al. (2009). Additionally, attention was given to residual correlations greater than.10 (Maydeu-Olivares et al., 2018). SEM was performed with the programs R *lavaan* (Rosseel, 2012) and *semTools* (Jorgensen et al., 2021).

As part of the internal structure study, measurement invariance and reliability were analyzed (American Educational Research Association, American Psychological Association, and National Council on Measurement in Education, 2014). Measurement invariance was measured with a bottom-up approach, from an unconstrained model to a strongly constrained model (Stark et al., 2006). Thus, we tested an unconstrained model (configurational invariance) and continued with successive restrictions applied to factor loadings, thresholds (metric invariance), and intercepts (scalar invariance). Given the sample size (> 300; Chen, 2007), the invariance criterion used were CFI < 0.010, SRMR < 0.030, and RMSEA < 0.015 (Chen, 2007). Age and gender were chosen as possible phenotypical sources of measurement variability since both variables are usually involved in studies of invariance in psychosocial measures. The campus of the study was chosen as a context variable where the degree of clustering of shared experiences may be present among students within the campus.

The reliability estimate for the score was made for congeneric measures, with the omega for categorical variables (ω; Green and Yang, 2009). For reference, the alpha coefficient (α) was also estimated. For both coefficients, confidence intervals were created at the 95% level (Bootstrap simulation method; Kelley and Pornprasertmanit, 2016). The R *MBESS* package was used (Kelley, 2019).

##### Description and Relationship With Other Variables

The description of the direct score of the SNA-6 was made by evaluating its fit to one of the seven distributions of the Pearson system (Ord, 2006). The R *Pearson DS* program (Becker and Klößner, 2021) was used. The relationships of the SNA-6 scores with other variables were examined using linear correlations and multiple line regression controlling for the effects of sex and age. The scores used were the simple sum of the items of the criterion variables (BSSS and CESD-7) and the latent factor of the SNA-6.

## Results

### Response Biases

The *G*_+_ scores ranged between 0 and 68 (*M* = 4.75. Md = 1, Q1 = 0, Q3 = 6, IQR = 6). For the identification of infrequent cases, cases above the cutoff point based on Tukey’s whiskers (Q3 + 1.5 * IQR = 15; Zijlstra et al., 2011, 2013) were chosen. Therefore, 278 participants with *G*_+_ ≥ 15 had an infrequent response pattern. With respect to *D*^2^, with a cutoff point of 16.81 (gl = 6, *p* < 0.01), 278 cases with a score *D*^2^ > 16.81 were identified and removed from the database. *D*^2^ between 16.41 and 73.37 (M = 6, Md = 3.19). The two identification criteria converged on *r* = 0.75 (*p* < 0.01), with an agreement of 209 true positives (*χ^2^* = 2107.1, gl = 52, *p* < 0.01, *Cramer-V* = 0.83). The effective sample for the following analyses was 2,789 participants (cases removed in total: 209, 6.9%).

### Item Analysis

#### Univariate Description

Table 2 shows the results. The distribution of responses was positive asymmetric, with a higher density around the infrequent expression of behavior dependent on SNs. The response difference in the items was statistically significant (Friedman -*χ^2^* = 485.32, *p* < 0.01) but trivial in size (*Kendall-W* = 0.035). The association with sex was approximately zero and not statistically significant; in the same way, in the associations with age, semester, and school, the exact variation of the associations was between 0.0001 and 0.003, none with statistical significance. Multivariate normality was rejected (Henze-Zirkler β = 382.3929, *p* < 0.01).

**TABLE 2:** Descriptive properties for the social networks addiction (SNA)-6 items.

	Correlations									
	Sna1	Sna2	Sna3	Sna4	Sna5	Sna6	Sex	Age	Sem	Campus
Sna1	1						0.00	0.00	0.00	0.00
Sna2	0.59	1					–0.03	0.00	0.00	0.00
Sna3	0.58	0.76	1				0.00	0.00	0.00	0.00
Sna4	0.56	0.68	0.73	1			–0.03	0.00	0.00	0.00
Sna5	0.51	0.57	0.61	0.62	1		–0.03	0.00	0.00	0.00
Sna6	0.59	0.69	0.72	0.74	0.65	1	–0.06	0.00	0.00	0.00

**Frequencies of options**										
	**Sna1**	**Sna2**	**Sna3**	**Sna4**	**Sna5**	**Sna6**				

Op 1	1689	1902	1828	1871	1924	2217				
Op 2	838	724	732	677	533	494				
Op 3	336	257	295	295	299	178				
Op 4	88	75	79	94	140	69				
Op 5	47	40	64	61	102	40				

**Statistic descriptive**										
	**M**	**SD**	**Sk**	**Ku**	**CvM**					

Sna1	1.61	0.84	1.43	1.93	54.98					
Sna2	1.48	0.78	1.77	3.24	75.60					
Sna3	1.53	0.82	1.69	2.84	68.67					
Sna4	1.52	0.82	1.70	2.86	72.14					
Sna5	1.54	0.90	1.70	2.23	81.43					
Sna6	1.34	0.71	2.45	6.64	112.05					

*All associations were statistically non-significant. Sna: items from the social network addiction scale (SNA-6). Op: response options: never (op1), rarely (op2), sometimes (op3), almost always (op4), always (op5). Sk: skewness coefficient. Ku: kurtosis coefficient. CvM: Cramer von Mises normality test. Sem: semester.*

#### Structure of the Response Options

The results are presented in Table 3. The *χ^2^* adjustment statistic to the PCM indicated that items 2, 3, 4, and 6 work appropriately with this model (*p* > 0.10); items 1 and 5 did not (*p* < 0.01). The Out-MSQ and In-MSQ values for each item were similar, greater than 0.50 and less than 1.30. According to what is suggested in this type of modeling (Wright and Linacre, 1994; Linacre, 2002a), based on Out-MSQ and In-MSQ, the items can be considered in a productive measurement range. The MSQ values in items 1 and 5 were also in an acceptable range. The discrimination of the items was high (> 0.49; between 0.50 and 0.82), and they were moderately similar. Additional and global indicators of fit of the model were separation reliability = 0.76, observed variance (squared standard deviation) = 2.48, and mean square measurement error (model error variance) = 0.58. Taken together, of all the results presented in this paragraph, the PCM model seems to be sufficient to parametrically model the structure of the response options.

**TABLE 3:** Partial Credit model Fit for ítems of SNA-6.

	PC Fit results (Partial Credit model)					Parameter items from PC model					
	*χ^2^* (df: 1757)	Out-MSQ	Out-t	In-MSQ	In-t	Disc.	Loc	Thr_1_	Thr_2_	Thr_3_	Thr_4_
Sna1	2250.57*	1.28	7.84	1.24	6.92	0.50	0.71	–2.51	–0.38	1.87	3.86
Sna2	1435.51	0.81	–5.18	0.79	–6.38	0.73	1.24	–1.89	0.07	2.16	4.64
Sna3	1276.95	0.72	–8.30	0.69	–9.98	0.79	0.83	–2.05	–0.21	1.99	3.62
Sna4	1375.67	0.78	–6.22	0.75	–7.68	0.75	0.92	–1.94	–0.25	2.12	3.77
Sna5	1900.47*	1.08	1.77	1.11	3.14	0.60	0.64	–1.59	–0.67	0.91	3.93
Sna6	963.96	0.54	–9.92	0.63	–10.57	0.82	1.49	–0.99	0.43	2.46	4.09

Sna: items from the social network addiction scale (SNA-6). Thr_:_ threshold parameter. Loc: location parameter. Disc.: discrimination parameter. In-t, Out-t: statistical test for In-MSQ and Out-MSQ. df: degree free. *p < 0.05.

##### Frequency of Categories

All response options met the criteria (*n* ≥ 10; Table 2). Particularly, the response option where the lowest distributional density occurred (*always* option) was between 4 and 6.4 times greater than the criterion *n* ≥ 10. The prevalence of response ranged from 1.3 to 6.4% for each item. Complementarily, the possible floor and ceiling effects were observed, defined as the high percentage in the highest or lowest response option, respectively. Considering the frequencies of the options (Table 2), the first two response options exceed 15% prevalence, and the first option had a very high prevalence (% > 55), indicating a floor effect in each item of the SNA.

##### Monotonic Increase in Response Options

**FIGURE 1:**
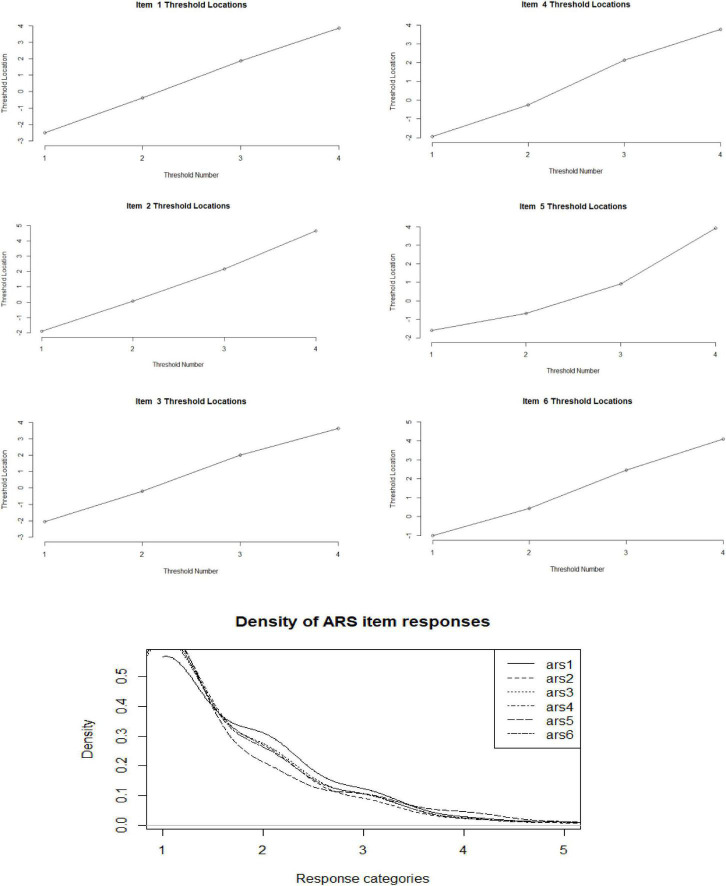
Evaluation of threshold monotonicity and distributional regularity (SNA-6 scale).

In Figure 1 – 1.1, the thresholds show a monotonic and regular advance (horizontal axis) in the range −3 to + 4 of the latent attribute (vertical axis). This regularity is linear and is slightly altered in items 4 and 5, but they do not seem to suggest a substantial departure from the observed linear increase. In conclusion, this structural criterion was met.

##### Regular Observation Distribution

In Figure 1 – 1.2, the distributional characteristics are shown by kernel densities (Gaussian smoothing, adjusted = 3). Similar characteristics are shown in all the items in the trend toward unimodality of the responses, in the prevalence of the first two adjacent categories, in the long tails to the left, and in the generally strong distributional similarity in all the items. According to these results, the item distributions were regular.

##### Ordering Thresholds (Step Calibrations Advance)

**FIGURE 2:**
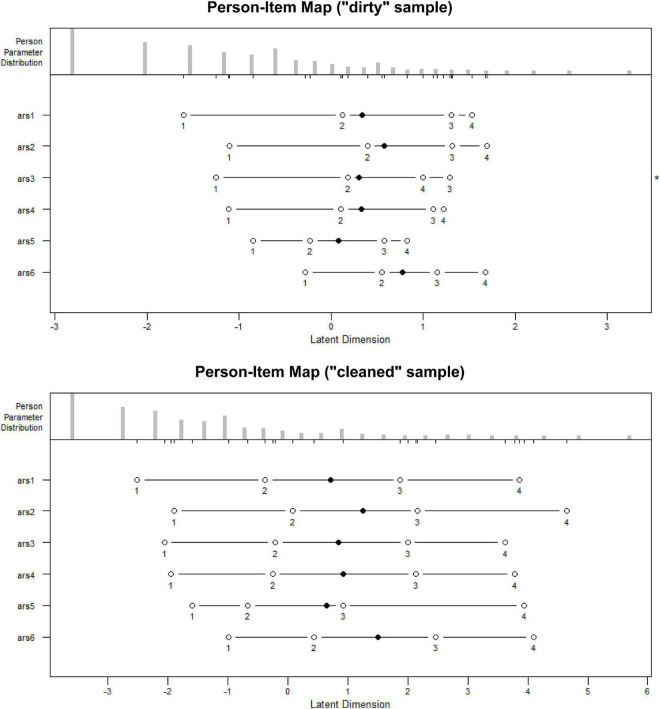
Orderings thresholds: clean and “dirty” sample (SNA-6 scale).

In Figure 2, the ordering of the k – 1 threshold (*k* = number of response options) in two moments of the data is presented: before (Figure 2.1) and after (Figure 2.2) the removal of potentially biased data. A strong inconsistency occurred in the data with potential responses with insufficient effort, in the first two thresholds, and the last two thresholds. This inconsistency of the first two options involved variable and wide spacings, while the inconsistency in the last two thresholds was small. The disorder is also observed in item 3, and little distinction is observed in the last two thresholds of item 4. In contrast to the above, in the lower subgraph (Figure 2.2), consistency was observed in the ordering of the thresholds in an order expected for the structure of the items. In conclusion, this criterion was met.

##### Step Difficulties Advance: At Least 1.0 Logits, but Less Than 5.0 Logits

The average difference, deduced from the thresholds presented in Table 3 (Thr_1_ to Thr_4_), between each threshold was, from 1 to 4, −1.66 (min = −2.13, max = −0.92), −2.09 (min = −2.37, max = −1.5) and −2.06 (min = −3.02, max = −1.62), respectively. All these values are below the maximum (< 5) and above the minimum cutoffs (> 1). Thresholds 1 and 2 of item 5 were the exceptions, but this discrepancy with the criteria can be considered insubstantial for practical purposes; therefore the criterion was considered to be fulfilled. Altogether, this feature can be considered complete. According to the results in this section, the hypothesis on the structure of response options is fulfilled.

### Descriptive and Association With External Variables Results

#### Internal Structure

##### Dimensionality

The one-dimensional model was rejected due to its statistical significance (null model: substantive or theoretical model), but the approximate fit indices were satisfactory because they met the criteria (Table 4; one-dimensional model heading). The WRMR was slightly higher than the criterion (WRMR > 1.0). Factor loadings tended to be greater than 0.80 (except item 1). On the other hand, in the estimation of the model with the method factor (F_met_), the first adjustment required setting a parameter to achieve adequate identification; thus, item 2, which obtained a very low load on F_met_ in the method factor model, was set at 0.0. With this added specification, the fit improved in all indices (i.e., random intercepts, RI; Table 4, heading one-dimensional model with method factor), and they were superior to those in the model without the method factor. The RI factor loads were not equal and were below.45 (between 0.168 and 0.421). The percentage of variance at the item level, attributed to the RI factor, varied between 2.8% (item 3) and 17.7% (item 6), and at the factor level, approximately 4.5% of the variance in the substantive factor was due to method variance, an amount that can be considered low. Controlling for the effect of the method, the factorial loads of the substantive factor remained high and were above.70. On the other hand, the modification indices (Md = 3.80, min = 0.015, max = 35.63) were relatively small, and although some were statistically significant, the standardized correlated residuals ranged between −0.05 and 0.07 (Md = −0.001) and were trivial in size. Therefore, no specification based on modifying indices was needed. The instrument dimensionality hypothesis (one dimension) is accepted.

**TABLE 4:** Unidimensional and unidimensional with method factor models.

	Unidimensional	Unidimensional + factor method	
	F	F	Fmet
Sna1	0.750	0.717	0.217
Sna2	0.895	0.945	0.000 (fixed)
Sna3	0.920	0.906	0.168
Sna4	0.902	0.843	0.321
Sna5	0.822	0.741	0.392
Sna6	0.938	0.857	0.421
Var	0.563	1 (fixed)	0.047
Reliability	0.91	0.825	0.086
WLSV-*χ^2^* (df)	53.540 (9)**	6.927 (4)	
CFI	0.999	1.000	
RMSEA (90% CI)	0.042 (0.032,0.053)	0.016 (0.00,0.036)	
SRMR	0.018	0.008	
WRMR	1.17	0.421	

Sna: items from social network addiction scale (SNA-6). F: factor loadings for unidimensional factor. Var: Factor Variance. Fmet: Method factor (random intercepts). Reliability: omega coefficient. **p < 0.01.

##### Measurement Invariance

To measure invariance across the age of the participants, the groups were recategorized to compare balanced groups of 14- to 16-year olds (*n* = 1,353) and 17- to 21-year olds (*n* = 1,428). In Table 5, the results indicate equivalence across sex, age, and campus groups. It was maintained up to the scalar invariance level (this is, equality of intercepts). As a consequence of the above, the hypothesis of the invariant properties of internal structure is accepted.

**TABLE 5:** Measurement invariance results for the SNA-6 scale.

	WLSMV *χ^2^*	*C* *F* *I*	RMSEA (CI 90%)	SRMR	Differences		
					Δ_*CFI*_	Δ_*RMSEA*_	Δ_*SRMR*_
**Sex**							
Configurational (df = 18)	56.364	1.00	0.039 (0.028,0.051)	0.019	–	–	–
Thresholds + loadings (df = 35)	104.61	0.999	0.038 (0.030,0.046)	0.022	0.001	0.001	–0.003
Intercepts (df = 40)	118.548	0.999	0.038 (0.030,0.045)	0.022	0.00	0.00	0.00
**Age**							
Configurational (df = 18)	59.22	0.999	0.041 (0.029,0.052)	0.019	–	–	–
Thresholds + loadings (df = 35)	87.85	0.999	0.033 (0.024,0.042)	0.023	0.00	0.008	–0.004
Intercepts (df = 40)	91.66	0.999	0.030 (0.02,0.039)	0.023	0.00	0.003	0.000
**Campus**							
Configurational (df = 18)	68.17	1.00	0.030 (0.014,0.044)	0.020	–	–	–
Thresholds + loadings (df = 35)	132.15	1.00	0.017 (0.00,0.029)	0.024	0.00	0.013	–0.004
Intercepts (df = 40)	152.46	1.00	0.016 (0.00,0.027)	0.024	0.00	0.001	0.000

##### Reliability

The coefficients ω (0.91; 95% CI = 0.90,0.92; se = 0.004) and α (0.91; 95% CI = 0.90,0.92; se = 0.004) of the scores were nearly equal. The standard error of measurement in the total sample was 1.23 (SD = 4.10); in men and women, it was 1.32 (SD = 4.43) and 1.12 (SD = 3.74), respectively.

### Association With Other Variables, Descriptive, and Difference Results

The distribution of the SNA-6 score was apparently not unimodal (D test = 0.07, *p* < 0.001). The parametric distribution with which it seems to fit was Pearson Type II (i.e., beta symmetric), since the model selection criteria were more minimized (log likelihood = −2.05; Akaike Information Criterion, AIC = 10.11, Bayes Information Criterion, BIC = 8.27) than the other distributions of the Pearson system, from 0 to VII (log likelihood < −3.36; AIC < −7.47; BIC > 12.28). The parameters for this distribution are shape (skewness) = 0.67, location = 2.05, and scale = 7.25.

On the other hand, age (*B* = −0.19; β = −0.05, 95% CI = −0.18,0.07; *p* = 0.003) and sex (*B* = 0.44; β = 0.15, 95% CI% = −0.25,0.36; *p* = 0.004; men coded as “2”) produced variability on the latent variable of SNA-6 but a size that can be considered weak and moderate, respectively (β < 0.20: weak, β < 0.50 moderate; Acock, 2014, p. 272). Regarding the campus, only campus 241 was statistically significant, and the variability can be considered weak (*B* = 0.47; β = 0.05, 95% CI = −0.34,0.45; *p* = 0.01).

In obtaining evidence of validity with other constructs, the latent linear correlations of the SNA-6 score with sensation seeking and depressive symptoms (BSSS and CESD-7, respectively) are shown in Table 6. The size of these latent correlations was small, and they were essentially unchanged after controlling for the effects of sex and age. All correlations were positive; accordingly, the hypothesis of association with depressive symptoms and sensation-seeking is accepted.

**TABLE 6:** Latent correlations and descriptive statistics for scores.

	SNA – 6	BSSS	CESD-7
**Correlations**			
SNA-6	1	0.24**	0.27**
BSSS	0.25**	1	0.25**
CESD-7	0.25**	0.25**	1
**Descriptives**			
M	9.05	24.88	13.25
SD	4.1	8.49	4.61
Sw	2.06	–0.28	0.22
Ku	5.19	–0.71	–0.54

*Below the diagonal: latent correlations, not controlling for sex and age. Above the diagonal: latent correlations, controlling for sex and age. SNA-6: social network addition score. BSSS: sensation seeking score. CESD-7: depressive symptom score. Sw and Ku: skew and kurtosis coefficients.*

## Discussion

The psychometric properties of the SNA-6 (Salas-Blas et al., 2020) were studied in the Mexican context, a measure of addiction to SNs created in Peru with potential psychometric characteristics that allow its scores to be interpreted from an emic framework. The study focused on the content of the items, the internal structure, and the relationship with external variables. As an additional note with methodological implications, the analysis performed began with the identification of participants who likely provided C/IE responses. The prevalence obtained from C/IE was 6.9%, which is not far from the wide range of prevalence reported in other studies (Arias et al., 2020). Although it seems small with respect to the participant sample size (initially, *n* = 2,998), the literature has consistently shown the existence of this C/IE response pattern in measures based on self-report surveys and its consequences on psychometric and non-psychometric results (Zijlstra et al., 2007, 2011, 2013; Meade and Craig, 2012; Meyer et al., 2013; King et al., 2018; Arias et al., 2020). Even with 2.5% random responses, the relevant estimates for the psychometric interpretation of the scores are inflated, something that particularly occurs in highly skewed and low prevalence samples (Cornell et al., 2012; Huang et al., 2015; Jia et al., 2018; King et al., 2018), as occurs in the distribution of SNA-6 scores. A practical implication of these results on the quality of the SNA-6 scores is that adjustments to the reliability estimates may be required with available methods (e.g., Fong et al., 2010), but the detection and removal of cases may be a more common, parsimonious, and secure solution when the data are in the hands of the user or researcher.

In the analysis of the SNA-6 response categorization, two things can be highlighted. First, this structure of response options worked appropriately in the present sample because none of the quality indicators proposed by Linacre (1999, 2002a) were challenged. It is also true that some of the items and thresholds did not meet these exact criteria. However, these discrepant values were not severely distanced from the criteria, and for practical purposes, they can still be considered within the chosen criteria. Even with these optimal results, some issues seem to require attention. The first of these is the floor effect found for all items, expressed in very high values (approximately 50% response in the first category, the lowest). As a consequence, the last two answer options were infrequently chosen. In health status measures, the criterion is generally > 15% to identify a significant floor or ceiling effect (Terwee et al., 2007), but in psychosocial measures, there is not a consensus or disseminated criterion in the scientific community. Therefore, the severity or rationality of the floor effect found must be evaluated in relation to the construct and expected use as defined in the present study. In this sense, the SNA measures a construct characterized by the greater intensity of the attribute and linked to inappropriate or maladaptive behaviors; since the participating sample was chosen to represent the distribution of this characteristic in the general community, a strong distributional asymmetry is expected, with greater density in the low scores. As corroborated in the descriptive and distributional analyses at the item level, what happens is precise that the responses of excessive dependence to SNs are not intense but rather low dependence. In summary, the potential floor effect problem is associated with the distributional asymmetry of the scores (Koedel and Betts, 2010), and it is not a constructed problem. Usually, attention is given to problems resulting from the floor effect, produced by the asymmetry of the score distributions (Koedel and Betts, 2010), such as the modeling of group differences (Šimkovic and Träuble, 2019). However, with modern robust analysis methods, this is not always a problem, especially when models such as generalized linear models are used (Šimkovic and Träuble, 2019). Moreover, the scores with asymmetric distribution of the SNA can be modeled with gamma, beta-binomial, and other distributions (Šimkovic and Träuble, 2019).

The second aspect that we highlight in the analysis of the SNA-6 response categorization is that although the results of the SNA-6 response options structure were adequate with the Linacre (1999, 2002a) criteria, the user can still decide whether the response scaling should be optimized, for example, toward a scaling with fewer response options due to the floor effect. In this situation, three options seem to be available to make modifications without losing the ordinal nature of the response and maintain the ordering of the thresholds (Zhu et al., 1997). There is evidence that indicates that the results of optimizing the response categorization, made from a Rasch approach (as applied in the present study), can be stable and reproducible in similar samples (Zhu, 2002). However, since the five SNA response categories remained optimal, the results should be replicable in similar samples.

As an additional note regarding the evaluation of the structure of the response options of the SNA, although the predominant tendency of the items was to be below 1.0 in MSQ, it did not decrease the quality of the attribute measurement. Because the instrument was constructed with techniques derived from the classical theory test, high response consistency is expected and used to select the items in the study by the SNA authors (Salas-Blas et al., 2020). Within the Rasch framework, this usually points to redundancy in responses and a highly predictable pattern of responses; however, this characteristic is not always a problem (Wright and Linacre, 1994; Linacre, 2002b).

The item-level analyses also included the association with external variables, to provide utility for construct interpretation at the level of specific behaviors (i.e., items; Koller et al., 2017), in relation to testing fairness and the possible emergence of differential item functioning (DIF), by showing possible context dependencies or phenotypic traits (e.g., sex and age), as well as for psychometric hypothesis formation at the level of scores and item selection. Here, the association between the SNA-6 items and the chosen variables was essentially zero (sex, age, study semester, and campus), and suggests that the specific contents do not covary in a magnitude that may indicate a differential functioning or multilevel approach to understanding social network addiction in the Mexican sample.

The SNA was unidimensional and showed high similarity in its factorial loadings. This replicates the result of the original study in the Peruvian sample (Salas-Blas et al., 2020). Factor loadings significantly exceeded.65, and the fit was satisfactory at a close high fit level (Maydeu-Olivares, 2017) and much more satisfactory for the random intercept model (RIFA model). Because the RIFA showed an excellent fit, one may wonder exactly the intrinsic mechanism of this RI factor. The RIFA model captures a wide range of method effects (Podsakoff et al., 2003; Steenkamp and Maydeu-Olivares, 2021), and the specific origin of this source of variability cannot be specified without an analysis of the characteristics of the instrument and participants examined in the sample. Due to the high fit obtained from the one-dimensional model without including RI (RMSEA ≤ 0.05, SRMR ≤ 0.05, and CFI ≥ 0.95; Maydeu-Olivares, 2017), the interpretation of the SNA scores can exclude the RI factor (Steenkamp and Maydeu-Olivares, 2021), and the one-dimensional model without RI can be accepted as a reasonable representation of the SNA in the Mexican sample.

The apparent optimal quality of the SNA in the Mexican sample was also enhanced by the significant comparisons between groups that can be made, specifically between groups based on sex, age, and study establishment. Indeed, the SNA can give equivalent results for its structural properties because the evaluation of the measurement invariance was satisfactory. Because both biological attributes seem to be common variables investigated to examine the variability of dependency behaviors, comparisons according to sex and age would not be biased due to the structural properties of SNA-6 in the Mexican sample. This result was similar to that of the original SNA-6 study.

Regarding the measurement invariance, the levels reached suggest that the attribute can be measured equivalently between the groups compared. This constancy of the evaluated parameters (i.e., factorial faces, dimensionality, and intercepts) reduces the interpretation of the group differences toward the absence of this equivalence or the differential functioning of the items.

Regarding the distributional form, the density function that can best describe the SNA-6 score by population is Pearson Type II, a special case of the beta distribution. This means that the behavior measured by the SNA-6 shows a higher density in the lower areas of the scores, that is, with lower intensity of the attribute. Due to the nature of the construct measured by the SNA-6, a statistically normal distribution cannot be assumed, but a strongly asymmetric one can be. This does not represent a problem for the theoretical understanding of the construct in the population, since the normal distribution is realistically unlikely (Joo et al., 2017), and precision is required to describe characteristics with high distributional skewness (Trafimow et al., 2019). A practical implication is that the generation of scales must account for not only the mean and standard deviation but also, and at least, distributional skewness (Trafimow et al., 2019). Additionally, a floor effect of the scores is likely (Koedel and Betts, 2010), as discussed above.

Statistically significant differences were detected when comparing the data by age (lower ages scored slightly more than the higher ages), which partially confirms what was found by Salas-Blas (2019a). Likewise, the data are different when compared by sex (men have higher scores than women), which confirms the findings of Salas and Escurra (2014) and Müller et al. (2016). In both results obtained, it is important to highlight that the variability of the SNA-6 due to sex and age can be considered small and moderate, respectively, an issue that is quite frequent when psychological variables are related to biological variables.

It should be noted that these contradictory data on trends in behavioral addictions by age and by sex may be related to sociodemographic variables such as the characteristics of the city in which the participants reside, access to the internet, the place where they connect (home or public cabins), the technological facilities they have access to and some issues related to family functioning; a possible more systematic analysis of the studies carried out could allow us to determine which sociocultural variables could be conditioning one or the other result.

In the evidence of the relationships of SNA with other constructs, weak positive linear associations were found with the attribute of seeking sensations and symptoms of depression, and these were in a positive direction, a fact that confirms what was theoretically proposed. This size of the association seems to be common because with the sensation-seeking score (BSSS), the associations of constructs and behavioral criteria tend to be small (Hoyle et al., 2002; Lorca and Sanz, 2003; Stephenson et al., 2007) and therefore weak but statistically significant. Some studies that found these links do not contain the calculation of the strength or magnitude of the effect, so it is difficult to make comparisons. The relationship of these variables with the SNA-6 probably sets a framework of the expected size, since this phenomenon is observed in many correlational studies in psychology in which the magnitude of the relationship is low but statistically significant. (a phenomenon that could be important to study more carefully in the future). Together, these associations support the interpretation of the SNA-6 scores regarding their theoretical link with sensation seeking and depression symptoms.

Regarding the limitations of the study, no answers were obtained on the intensity of the use of SNs, and therefore, the link of this variable with dependent behavior could not be evaluated. Likewise, the representativeness of the population is not guaranteed, since the sample came from a single Mexican state, and therefore, a generalization cannot be made toward the total Mexican population. Studying the variables with self-report instruments can generate reasonable doubts about the results.

## Conclusion

The present study provides strong evidence of the validity and reliability of the SNA-6 questionnaire (Salas-Blas et al., 2020) in the Mexican context. These satisfactory properties included (a) an adequate structure of the response options, with potential for improvement by reducing the number of response options; (b) a replicable unidimensionality of the scores with respect to the study in Peruvian adolescents; (c) appropriate reliability values for screening assessments; (d) coherent theoretical relationships with measures of depressive symptoms and sensation seeking. Although the study was conducted in a representative sample of Mexico, the results obtained can be taken as reference values for contrast and support possible conclusions about the replicability of the present results. Given the convergence with studies in other countries (e.g., Peru and Brazil), the SNA-6 can be a useful tool to investigate SN addiction in Latin American countries in general and Mexico in particular.

## Data Availability Statement

The raw data supporting the conclusions of this article will be made available by the authors, without undue reservation.

## Ethics Statement

The present research was approved by the Commissions of Research, Ethics and Biosafety (Comisiones de Investigación, Ética y Bioseguridad), Hospital Infantil de México Federico Gómez National Institute of Health. Written informed consent to participate in this study was provided by the participants’ legal guardian/next of kin.

## Author Contributions

CM-S designed the study and performed the statistical analyses. BP-A acquired and validated the data. ES-B and BP-A contributed to the interpretation of the results. CM-S, ES-B, and BP-A approved it for publication. FT-T contributed to the writing, review, and editing and funding acquisition. CM-S and FT-T contributed to the visualization, project administration, and supervision. All authors drafted the initial and final version of the manuscript.

## Conflict of Interest

The authors declare that the research was conducted in the absence of any commercial or financial relationships that could be construed as a potential conflict of interest.

## Publisher’s Note

All claims expressed in this article are solely those of the authors and do not necessarily represent those of their affiliated organizations, or those of the publisher, the editors and the reviewers. Any product that may be evaluated in this article, or claim that may be made by its manufacturer, is not guaranteed or endorsed by the publisher.

## Footnotes

1
https://datareportal.com/reports/digital-2021-global-overview-report
